# Hydrothermal Pretreatment of KOH for the Preparation of PAC and Its Adsorption on TC

**DOI:** 10.3390/ma16144966

**Published:** 2023-07-12

**Authors:** Shouqi Wang, Linkai Wu, Liangcai Wang, Jianbin Zhou, Huanhuan Ma, Dengyu Chen

**Affiliations:** 1 College of Materials Science and Engineering, Nanjing Forestry University, Nanjing 210037, China; wangsq@njfu.edu.cn (S.W.); 11220341@stu.lzjtu.edu.cn (L.W.); wangliangcai@njfu.edu.cn (L.W.); zhoujianbin@njfu.edu.cn (J.Z.); chendy@njfu.edu.cn (D.C.); 2 Joint International Research Laboratory of Biomass Energy and Materials, Co-Innovation Center of Efficient Processing and Utilization of Forest Resources, College of Materials Science and Engineering, Nanjing Forestry University, Nanjing 210037, China

**Keywords:** pinecone-based activated carbon, tetracycline, adsorption kinetic

## Abstract

The environment has been heavily contaminated with tetracycline (TC) due to its excessive use; however, activated carbon possessing well-developed pores can effectively adsorb TC. This study synthesized pinecone-derived activated carbon (PAC) with high specific surface area (1744.659 cm^2^/g, 1688.427 cm^2^/g) and high adsorption properties (840.62 mg/g, 827.33 mg/g) via hydrothermal pretreatment methods utilizing pinecones as precursors. The results showed that PAC treated with 6% KOH solution had excellent adsorption properties. It is found that the adsorption process accords with the PSO model, and a large amount of C=C in PAC provides the carrier for π-πEDA interaction. The results of characterization and the isothermal model show that TC plays a key role in the adsorption process of PAC. It is concluded that the adsorption process of TC on PAC prepared by hydrothermal pretreatment is mainly pore filling and π-πEDA interaction, which makes it a promising adsorbent for TC adsorption.

## 1. Introduction

Tetracyclines (TC) are extensively employed in veterinary, clinical, and pharmaceutical practices due to their exceptional antibacterial properties [1]. However, the release of tetracyclines into the environment, such as surface water, groundwater, and sediments [2], without proper degradation can contribute to the proliferation of tetracycline resistance genes and the emergence of tetracycline-resistant bacteria, leading to severe ecological damage [3]. The aqueous environment serves as a significant habitat for tetracycline, highlighting the importance of efficient wastewater treatment processes for the removal of tetracycline to safeguard the ecosystem [4].

The primary techniques used to eliminate tetracyclines from wastewater are biodegradation, chemical degradation, and physical degradation [5]. Biodegradation employs enzymes produced by microbial cells during metabolic activities to break down and eliminate antibiotics [6]. This approach is cost-effective, easy to operate, and does not result in secondary pollution. However, it has drawbacks such as a large footprint, long cycle time, low efficiency, and difficulty in effectively eliminating trace pollutants. Chemical degradation, on the other hand, utilizes chemical reagents to initiate a reaction with tetracycline, causing it to decompose or polymerize into new products [7]. This method is commonly used in wastewater treatment but is susceptible to producing secondary pollution, such as toxic by-products from the light Fenton process [8]. Physical degradation involves the physical treatment of water to separate tetracycline from it. Flocculation, membrane separation, and adsorption are the primary methods used for physical degradation [9]. Conventional tetracycline wastewater treatment techniques are associated with issues such as low degradation efficiency, high cost, and the presence of side reactions. The adsorption technique overcomes many of these drawbacks [10] and has the benefits of being easily accessible and having simple synthesis procedures [11]. The most commonly used adsorbents are metallic compounds [12], zeolites [13], activated carbon (AC) [14], etc. Among them, the importance of carbonaceous materials in water purification is irreplaceable [15]. Xiang et al. [16] demonstrated that various carbon-based adsorbents, such as carbon nanotubes, activated carbon, and graphene, are effective in removing tetracycline from water. Jia et al. [17] created a Co/Fe-MIL-100 adsorbent material that can remove nearly 100% of tetracycline at initial concentrations ranging from 10 to 40 mg/L, and still achieve an 82.38% removal rate at a tetracycline concentration of 60 mg/L. Ranjbari prepared novel ionic liquid-impregnated chitosan hydrogel beads (CS-TCMA) for the rapid adsorption of tetracycline and found that the maximum adsorption of TC on CS-TCMA was 22.42 mg/g [18].

Biomass-derived activated carbon possesses excellent adsorption performance due to its large specific surface area, abundant pore structure, and functional groups [19]. Because of its low cost and wide availability of raw materials, it has been widely used to remove tetracycline from water bodies. The preparation of biomass-activated carbon from agricultural and forestry wastes for the adsorption of tetracycline in water bodies has been widely based on scientific research and practical applications. However, from the existing research contents, the preparation of biomass-activated carbon is mainly based on rice husk [20], corn straw [21], and sugarcane bagasse [22] as raw materials, while the research and development of pine needles are mostly focused on pine needle biochar-based supercapacitor and the adsorption of heavy metal ions (Zn^2+^, Pb^2+^, Cd^2+^, etc.), and there is little involvement and research on PAC adsorption of TC. In China, pine towers and pine needles are widely available and inexpensive. Because of their well-developed pore structure and being rich in nitrogen and oxygen, their carbonized activated carbon can perform chemisorption in addition to physical adsorption, and the preparation process is simple and environmentally friendly, which has a broad prospect as a new type of efficient adsorbent.

Hydrothermal pretreatment is a promising technology that has attracted great interest among researchers in the treatment of biomass feedstocks due to its simplicity and relatively short operation time [23]. The hydrothermal pretreatment process mainly takes place during hydrolysis, dehydration, condensation, and polymerization reactions, and there are micro- or mesopores and abundant functional groups in hydrates [24]; there is little known about the preparation of PAC from hydrates.

The porous structure of biomass-activated carbon is closely related to biomass precursors and activation conditions [25]. This study utilized pinecones, a type of biomass, as the precursor, and employed hydrothermal pretreatment processes and pyrolysis to produce pinecone-based activated carbon (PAC). The optimal concentration of the activator was selected, and the adsorption characteristics of activated carbon under different pretreatment processes were determined and analyzed using SEM, BET, and FTIR characterization methods. By investigating the effects of PAC dosage, TC concentration, adsorption time, and temperature on the adsorption of tetracycline (TC), the study identified the optimal conditions for the adsorption of TC by PAC. The adsorption process was further examined using kinetic and isothermal adsorption models.

## 2. Experimental Materials and Methods

### 2.1. Experimental Materials

The raw material of pinecones used in this experiment was harvested from Kuandian, Liaoning Province. The raw material of pinecone (PC) was washed with ultrapure water and dried, and it was crushed to below 200 mesh. The KOH used in the experiments was analytically pure from Sinopharm Chemical Reagent Co., Ltd. (Shanghai, China); and tetracycline (C_22_H_23_N_2_O_8_, T829835-100g, CAS:60-54-8) was purchased from Shanghai Macklin Biochemical Co., Ltd. (Shanghai, China).

### 2.2. Experimental Methods

#### 2.2.1. Preparation of Activated Carbon

This study employed two pretreatment processes to produce pinecone-based activated carbon. In the first process, pinecone (PC) was mixed with deionized water and then loaded into a reaction kettle heated at 160 °C for 1.5 h in a muffle furnace. Subsequently, the PC was mixed and impregnated with KOH solution for 8 h. The mixed sample was ramped up to 900 °C at a temperature rise rate of 10 °C/min under N_2_ protection and held for 1 h to produce activated carbon, designated as FX-PAC (X represents the concentration of KOH-impregnated activated carbon). The second process involved mixing PC and KOH solution in a reaction kettle, which was heated to 160 °C for 1.5 h in a muffle furnace. The mixed sample was then raised to 900 °C under N_2_ protection at a heating rate of 10 °C/min for 1 h to produce activated carbon, designated as KY-PAC (Y represents the concentration of KOH-impregnated activated carbon). The resulting activated carbon samples were characterized and evaluated for their adsorption performance towards tetracycline under different conditions.

#### 2.2.2. Characterisation of Activated Carbon

The activated carbon microstructure was examined using a field emission scanning electron microscope from Japan. The specific surface area, pore size distribution, pore volume, and micropore volume of PAC were obtained using an Autosorb-iQ automatic specific surface area and pore size distribution instrument from the USA, with vacuum degassing at 300 °C for 900 min under liquid nitrogen at 77 K. The infrared spectra were measured by a Fourier infrared spectrometer from Bruker, Germany, and the functional groups’ qualitative and structural analyses were conducted based on the characteristic frequencies of the groups, including peak position, peak number, and peak intensity.

#### 2.2.3. Tetracycline Adsorption Experiments

**Figure 1:**
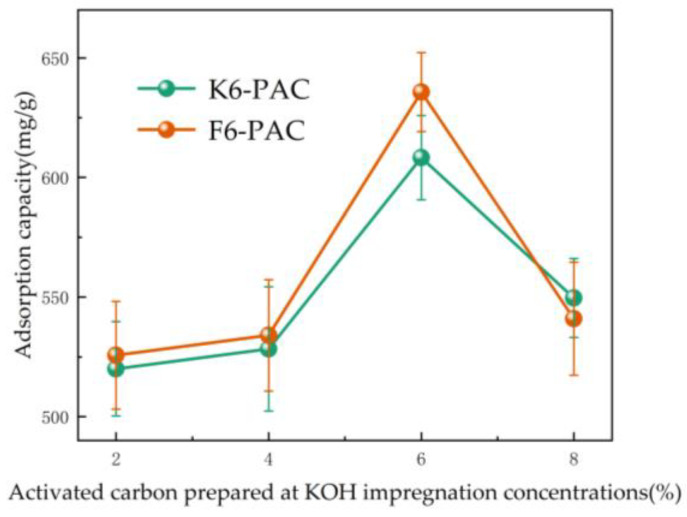
Adsorption capacity of activated carbon prepared at different KOH impregnation concentrations.

The tetracycline powder was weighed and prepared into 100 mg/L of TC solution. Different activated carbon samples were added sequentially and stirred at 313 K (40 °C) for 24 h; the absorbance of their supernatant was measured after filtration. The adsorption amount was calculated using an equation, and a line graph was plotted for the different KOH impregnation concentrations of the prepared activated carbons versus the adsorption amount $q_{t}$ (mg/g) as shown in Figure 1. The optimum PAC for TC adsorption was selected by comparing the adsorption amount $q_{t}$.

The following equation Is used to calculate the adsorption capacity:

$$q_{t}=\frac{(C_{0}-C_{e})V}{m}$$

where $C_{0}$ is the initial concentration of solute (mg/L), $C_{e}$ is the equilibrium concentration of solute (mg/L), V is the volume of solution (L), m is the mass of adsorbent (g), and $q_{t}$ is the amount of adsorption of the target pollutant by the adsorbent at time t (mg/g).

The adsorption capacity of PAC treated by impregnation with different KOH concentrations increased with the increase of KOH concentration (Figure 1), but the adsorption capacity of PAC decreased significantly when the KOH concentration reached 8%, which was due to the excessive ablation of activated carbon caused by the excess KOH. The adsorption capacity of PAC did not increase significantly when the KOH concentration increased from 2% to 4%, but when the KOH concentration increased to 6%, the adsorption capacity increased to its highest. Minor changes in the concentration of KOH can elicit huge differences in the adsorption capacity of PAC. Therefore, in this study, K6-PAC and F6-PAC were used as the main objects for structural analysis, and the adsorption mechanism was further investigated through adsorption reaction kinetics experiments. The present study focused on the structural analysis of K6-PAC and F6-PAC using various characterization methods and further investigation of their adsorption mechanisms through kinetic experiments.

#### 2.2.4. Adsorption Isotherms and Kinetic Models

In this paper, we investigate the adsorption characteristics of PAC on TC under different adsorption conditions of adsorption temperature, PAC dose, and adsorption time of TC.

(a) Adsorption Kinetic Model

To investigate the adsorption properties and mechanism of action of PAC, the pseudo-first-order kinetic model (PFO), pseudo-second-order kinetic model (PSO), W-M intraparticle diffusion model, and Boyd model were used for data fitting and analysis [26].

The adsorption kinetic models and equations are shown in Table 1.

(b) Adsorption Isotherm Model

The sorption isotherm plays a crucial role in estimating the maximum sorption capacity of an adsorbent [27].

The adsorption isotherms and equations are shown in Table 2.

## 3. Results and Discussion

### 3.1. Characterization Analysis of PAC

#### 3.1.1. Specific Surface Area and Pore Size Distribution of PAC

Quantitative analysis of the pore structure, including specific surface area, pore size, and micropore volume, is crucial in determining the adsorption performance of activated carbon. The specific surface area and pore size distribution of the PAC were measured using a fully automated specific surface area and pore size distribution analyzer [28], and the results are presented in Table 3. The findings indicate that the activated carbon obtained through the second pretreatment process had a greater specific surface area and a more developed micropore structure, which was attributed to the formation of micropores during the decomposition of KOH [29] in the PC and KOH impregnation pretreatment process followed by heating. In general, the adsorption efficiency of the adsorbent is strongly associated with its specific surface area, and as the specific surface area and micropore volume increase, the adsorption sites on the activated carbon surface increase as well, thus boosting the adsorption capacity [30].

**Figure 2:**
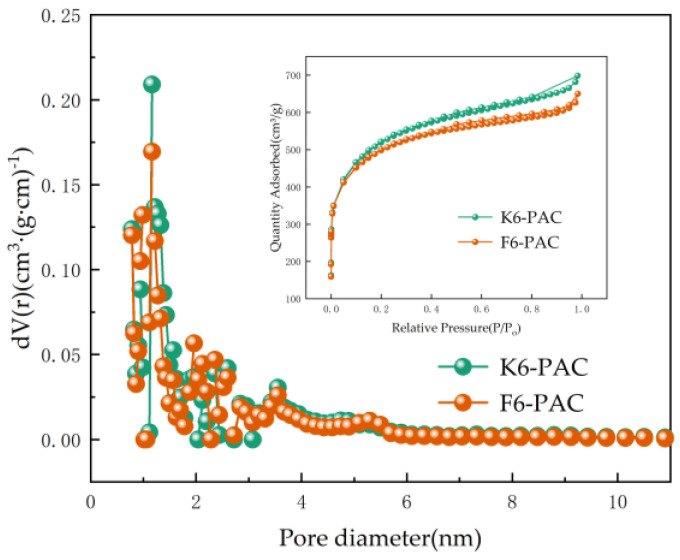
Adsorption-desorption isotherms and pore size distributions of PAC.

The N_2_ adsorption and desorption curve reflects the variation of adsorption amount with relative pressure (P/P_0_) at a certain temperature. As shown in Figure 2, the N_2_ adsorption-desorption of K6-PAC belonged to the type II isotherm, whereas F6-PAC was classified as a type IV isotherm with H3 hysteresis loops, which is associated with the abundance of mesopores inside the material [31].

**Figure 3:**
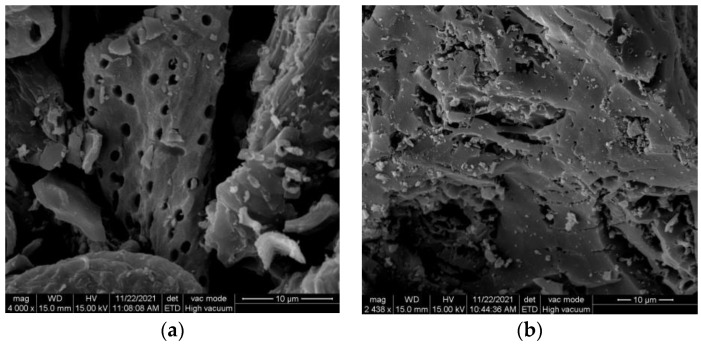
SEM of K6-PAC (**a**) and F6-PAC (**b**).

Microporous and mesoporous pores are predominant in PAC (Table 3), and the strong interaction between the adsorbent and the surface led to a rapid rise in N_2_ adsorption at lower relative pressure. The K6-PAC exhibited no distinct boundary between multilayer and monomolecular adsorption, and no capillary coalescence was observed, which combined with Figure 3 can be known to have a uniform pore channel distribution. In contrast, the F6-PAC displayed an isothermal inflection point near monolayer adsorption, where multilayer adsorption gradually formed with increasing relative pressure, and F6-PAC exhibited H3-type hysteresis loops and did not reach equilibrium when the relative pressure was close to saturation vapor pressure, indicating that its pore structure was incomplete and mostly slit-like [32]. These findings were consistent with the results of the pore size distribution characterization.

#### 3.1.2. SEM of PAC

Figure 3a demonstrates that K6-PAC has a relatively complete pore structure on its surface, and there are many cracks and roughnesses, which provide a large number of sites and facilitate the adsorption of TC. On the other hand, Figure 3b shows that F6-PAC has a rougher surface with small pores, many folds, and fine particulate matter floating on it, which is consistent with the capillary coalescence phenomenon mentioned in the N_2_ adsorption and desorption curve of F6-PAC. This indicates that the KOH and PC impregnation pretreatment process followed by heating plays a vital role in microporosity formation.

#### 3.1.3. FTIR of PAC

**Figure 4:**
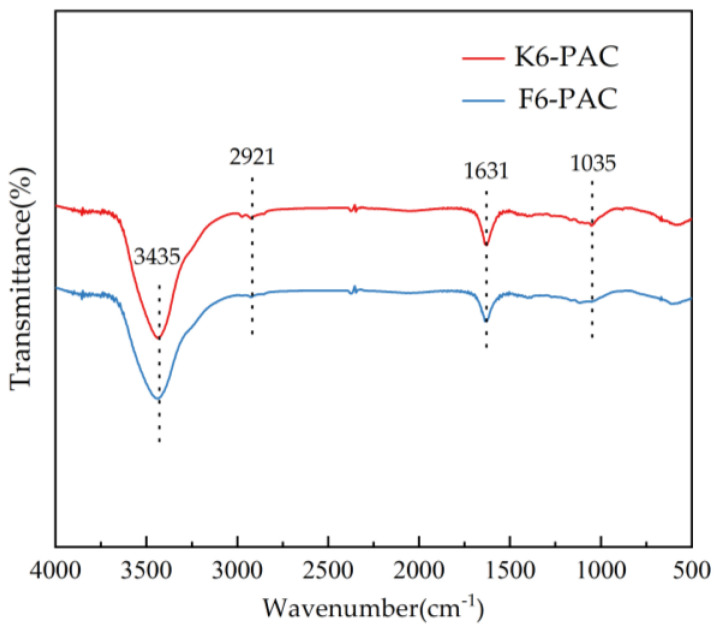
FTIR of PAC.

Figure 4 displays the FTIR spectra of activated carbon obtained by two pretreatment processes. The absorption peak positions were not markedly distinct, indicating that activation by KOH occurred in both pretreatment processes, albeit with different effectiveness. The hydroxyl group (-OH) [33] displayed characteristic absorption peaks at 3435 cm^−1^ in all PACs prepared through different pretreatment processes, attributable to the telescopic vibrational effect. Moreover, the characteristic peak at approximately 1630 cm^−1^ was attributed to the telescopic vibrations of C=O or C=C and absorption peaks at 2921 cm^−1^ and 1062 cm^−1^, which are attributable to the antisymmetric stretching vibrations and bending vibration of the C-H.

The π-π electron donor-acceptor (π-πEDA) interaction has been identified as the primary mechanism underlying the chemisorption of TC by activated carbon [34]. Based on the analysis presented above, it is evident that activated carbon contains C=C, which is one of the key carriers of the π-πEDA interaction [35]. As TC is an electron-deficient system, it can act as a π-electron acceptor in the reaction, and activated carbon activated by KOH possesses a strong electron supply capacity, resulting in a superior adsorption effect on TC.

### 3.2. Analysis of Tetracycline Adsorption on Activated Carbon

#### 3.2.1. Effect of Adsorption Time

**Figure 5:**
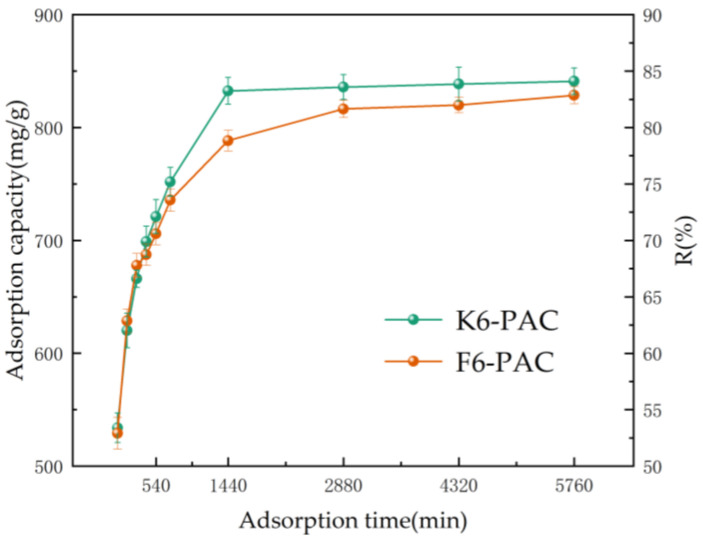
Adsorption capacity and R of PC at different adsorption times. (Adsorption temperature: 40 °C; $C_{0}:$ 100 mg/L; PAC dosage: 15 mg).

The initial concentration of TC ($C_{0}$) was 100 mg/L, the PAC dosage was 15 mg, and the adsorption temperature was set at 40 °C. The effect of adsorption time t on the adsorption of TC by K6-PAC and F6-PAC was investigated, and the results are shown in Figure 5.

The formula for the TC removal rate, R (%), is shown below:

$$R=\frac{C_{0}-C_{e}}{C_{0}}\times 100\%$$

where $C_{0}$ is the initial tetracycline concentration (mg/L), $C_{e}$ is the equilibrium tetracycline concentration (mg/L), and R is the removal rate (%).

In the initial 1440 min (24 h), the adsorption capacity of the two activated carbons increased gradually with time, and their TC removal rates reached 53.11% and 52.99% of the saturation adsorption capacity at 60 min, respectively, as shown in Figure 5. After 1440 min of adsorption, the rate of adsorption capacity increase slowed down, which is typical behavior for adsorption systems where the number of available adsorption sites on the activated carbon surface becomes limited over time. As can be seen from the graph, K6-PAC has reached equilibrium at 24 h, while F6-PAC reaches adsorption equilibrium at 48 h. This shows that the optimum adsorption time is 24 h for K6-PAC and 48 h for F6-PAC, with other operating parameters remaining unchanged.

#### 3.2.2. Effect of Adsorption Temperature

**Figure 6:**
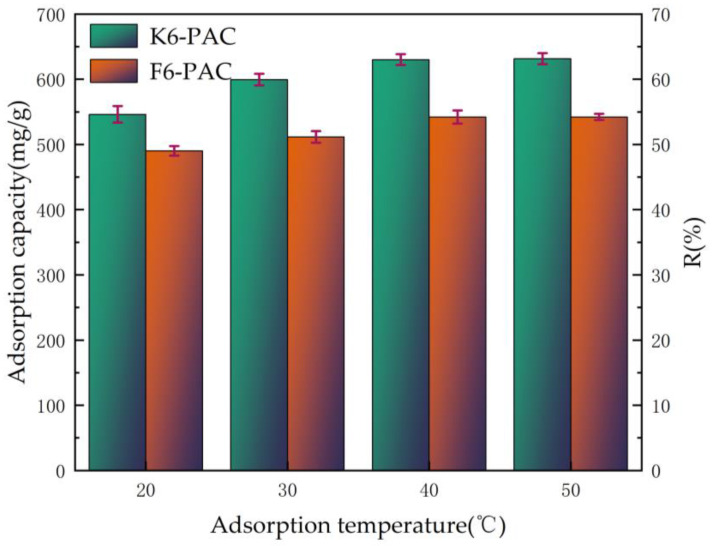
Adsorption capacity and R of PAC at different adsorption temperatures. (Stirring time: 1440 min; $C_{0}:$ 100 mg/L; PAC dosage: 15 mg).

The initial TC concentration $C_{0}$ was 100 mg/L, the dosage of PAC was added at 15 mg and stirred for 1440 min, and the effects of temperature conditions of 20 °C, 30 °C, 40 °C, and 50 °C on the adsorption of TC by K6-PAC and F6-PAC were investigated, respectively, as shown in Figure 6.

The gradual increase in the removal rate of tetracycline by K6-PAC and F6-PAC with increasing adsorption temperature was attributed to the expansion of the active centers of the adsorbent caused by the temperature increase, leading to an improvement in adsorption efficiency. At temperatures below 40 °C, the adsorption amount $q_{t}$ of K6-PAC increased slowly, with the removal rate R increasing from 56.43% to 63.05%. At 50 °C, the adsorption amount and removal rate of K6-PAC were 631.97 mg/g and 63.06%, respectively, and the adsorption efficiency at 50 °C was similar to that at 40 °C, which was due to a positive correlation between adsorption temperature and Brownian motion. The adsorption amount of F6-PAC increased slowly from 490.54 mg/g to 543.18 mg/g, and the removal rate increased from 49.05% to 54.31% before 40 °C. At 50 °C, the adsorption amount and removal rate of F6-PAC were 544.54 mg/g and 54.65%, respectively, and the same conclusion was reached. However, K6-PAC exhibited better adsorption performance than F6-PAC under the same adsorption conditions.

The results of the kinetic analysis showed that the equilibrium adsorption time and amount for both activated carbons were 96 h, 840.62 mg/g, and 827.33 mg/g, respectively, when the PAC dosage was 15 mg. Therefore, the optimal adsorption temperature was found to be 40 °C, assuming that other operating parameters remained constant.

#### 3.2.3. Effect of PAC Dose

**Figure 7:**
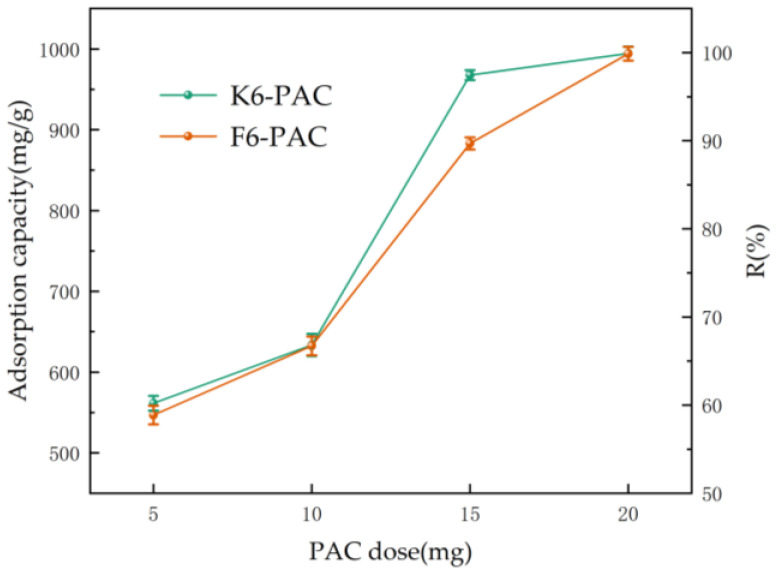
Adsorption capacity and R of PAC at different PAC doses. (Adsorption temperature: 40 °C; stirring time: 1440 min; $C_{0}:$ 100 mg/L).

In our study, 5 mg, 10 mg, 15 mg, and 20 mg of K6-PAC and F6-PAC were added to 100 mg/L of TC standard solution and adsorbed at 40 °C for 1440 min with stirring, and the absorbance of its upper clear layer was measured after filtration. The adsorption amounts and TC removal rates were calculated, as shown in Figure 7.

As the amount of activated carbon increased, both the adsorption capacity and TC removal rate increased as a whole. However, at a dosage between 5 to 15 mg, the adsorption effect of PAC did not meet the expected level due to the limited adsorption sites on the activated carbon surface. Once the dosage exceeded 15 mg, there was a significant increase in both the adsorption capacity and TC removal rate for PAC. When 15 mg of activated carbon was added, it served as the inflection point, beyond which the adsorption capacity and TC removal rate increased substantially.

When the activated carbon dosage increased from 10 mg to 15 mg, the average level of adsorption parameters for K6-PAC and F6-PAC increased by 54.21% and 39.55%, respectively, which were 1.54 and 1.40 times those of the 10 mg injection. However, compared to the adsorption parameters at 15 mg of carbon, the rates of increase were only 2.33% and 12.59%. This decrease in the rate of increase was due to the increased frequency of collisions between the activated carbon and the stirring bar and conical flask, leading to agglomeration and a slowdown in adsorption levels. Therefore, it can be concluded that the optimum amount of activated carbon is 15 mg, with all other operating parameters kept constant.

#### 3.2.4. Adsorption Kinetics

To further explore the mechanism of action of TC adsorption by PAC, PFO, PSO, W-M, and Boyd models were used to fit the adsorption data, and the relevant parameters are shown in Table 4.

**Figure 8:**
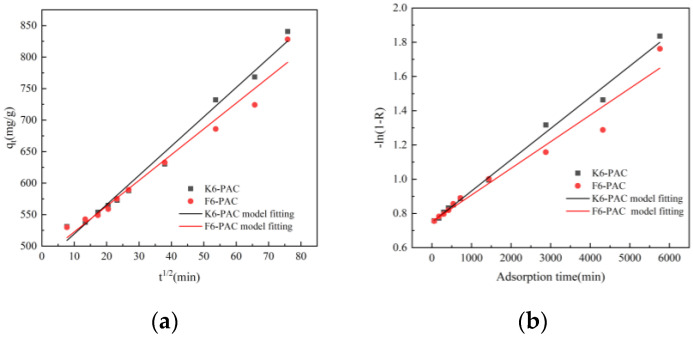
W-M (**a**) and Boyd (**b**) model fitting.

The PFO model was found to be unsuitable for describing the TC processes of PAC adsorption, as indicated by the low correlation coefficients ($R^{2}$) obtained for fitting the model to the adsorption processes of K6-PAC and F6-PAC. On the other hand, the PSO model showed higher correlation coefficients (0.9931 and 0.9896, respectively) for linear fits to the two activated carbons, indicating that this pseudo-second-order kinetic model was more appropriate for fitting the adsorption processes. Additionally, the equilibrium adsorption capacity of the PSO model was in better agreement with the experimentally measured values; this indicates that the adsorption process is mainly chemisorption. The W-M intraparticle diffusion model was also fitted to the adsorption process and gave correlation coefficients of 0.9865 and 0.9689, respectively, with $R^{2}$ larger than that of PSO. Therefore, the W-M intraparticle diffusion model is more suitable for describing the PAC adsorption process. However, the linear regression lines fitted to their data did not intersect the origin (Figure 8a), suggesting that the adsorption reaction rates were not exclusively governed by pore diffusion within the particles. According to the Boyd model in Table 4, the high $R^{2}$ linear correlation between K6-PAC and F6-PAC, coupled with the deviation of the fitted line from the origin, provides evidence for the involvement of liquid film diffusion in the adsorption mechanism. Therefore, it can be concluded that the adsorption of TC on PAC conforms to the pseudo-second-order kinetic model, and the entire diffusion process is accomplished through the synergistic control of membrane diffusion and intraparticle diffusion.

#### 3.2.5. Adsorption Isotherms

An adsorption equilibrium isotherm is a crucial tool that qualitatively characterizes the interaction process between the active adsorbent and the adsorbed object by describing the relationship between the equilibrium adsorption of activated carbon and the equilibrium concentration of the target pollutant. This relationship is established under the premise that the AC-TC solution has reached equilibrium in terms of the initial concentration of TC, constant temperature, and mass concentration.

**Figure 9:**
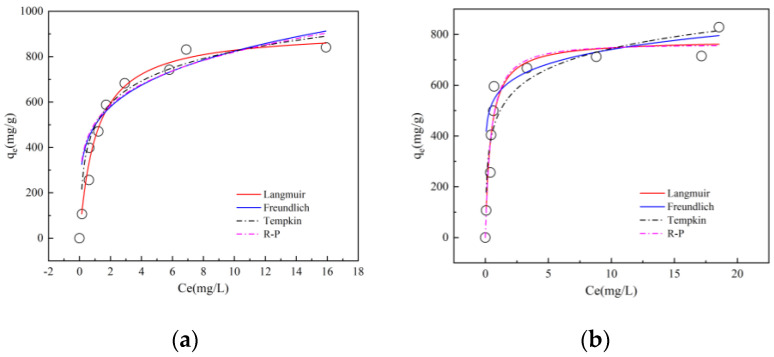
PAC isothermal adsorption model fitting ((**a**): K6-PAC; (**b**): F6-PAC).

The Langmuir isotherm, Freundlich isotherm, Temkin isotherm, and R-P isotherm were used to study the adsorption kinetics at different initial concentrations [36], as shown in Figure 9.

Based on the IUPAC classification, the isothermal adsorption curves indicate that the adsorption of TC on the surface layer of activated carbon saturates at much lower initial concentrations [37]. Figure 9 demonstrates that the equilibrium adsorption of TC, $q_{m}$, by both K6-PAC and F6-PAC, increases with increasing initial TC concentration $C_{e}$ until it reaches a maximum increase. This is due to the fact that at low initial TC concentrations, there are numerous adsorption vacancies on the PAC surface, but as the mass concentration of TC increases, the number of adsorption sites on activated carbon decreases. Once the adsorption vacancies are filled, TC diffuses into the interior of PAC, and adsorption nearly saturates after a certain period, leading to a decrease in its adsorption capacity.

Table 5 below shows the parameters associated with several model fits.

The Langmuir model assumes that the adsorption sites of adsorbents have the same affinity for pollutants, and a single molecule adsorption layer will be formed [38]. The Freundlich model is an important formula for studying multi-layer and heterogeneous adsorption behavior [39]. Table 5 shows that the Langmuir model affinity constant $k_{L}$ for F6-PAC (2.36153) is 2.56 times higher than that for K6-PAC, resulting in a rapid increase in the equilibrium adsorption of TC by F6-PAC with increasing initial TC concentration. The nonlinear coefficients $R^{2}$ for the Langmuir isotherm (0.91892~0.97599) and the correlation coefficients for the Temkin isotherm (0.91452~0.96682) were higher than the Freundlich model $R^{2}$ values (0.79174~0.86336), indicating that TC was uniformly adsorbed on the PAC surface. Both the Langmuir isotherm dimensionless constant $R_{L}$ and the Freundlich isotherm dimensionless constants $R_{L}$ and *n* fall within the range of favorable adsorption, with $R_{L}$ being close to 1, i.e., 0.0107 and 0.0042 for F6-PAC and K6-PAC, respectively, suggesting that the adsorption of TC by PAC is both favorable and irreversible. The Temkin isotherm fits better than the Freundlich isotherm, with $R^{2}$ values of 0.96682 and 0.91452 for the two carbons, and the model constants $b_{T}$ less than 1, indicating that the adsorption of TC by PAC is endothermic in the studied concentration range (0–100 mg/L). The R-P isotherm shows a better nonlinear correlation coefficient $R^{2}$ than the Langmuir isotherm, suggesting that the adsorption of TC by PAC results from the combined effect of monolayer and multilayer adsorption. However, the g parameters of the R-P isotherm are close to 1, i.e., 0.94905 and 0.91666 for F6-PAC and K6-PAC, respectively, indicating that the monolayer adsorption predominates in the TC adsorption process by PAC.

In summary, the TC adsorption process by pinecone-activated carbon involves both monolayer and multilayer adsorption [40], with monolayer adsorption predominating and the adsorption process being an irreversible endothermic reaction. Based on the analysis of TC adsorption on PAC, K6-PAC has a maximum adsorption capacity of 840.62 mg/g, which is greater than that of various adsorbents (Table 6).

### 3.3. Sorption Mechanism

In terms of the pore characteristics of activated carbon, the abundant and porous nature of its channels presents an excellent opportunity for maximizing the pore-filling effect. Likewise, numerous scholars and researchers widely concur that the pore-filling effect constitutes a vital mechanism for the degradation efficacy of activated carbon [46]. Based on the characterization of PAC, the PAC prepared after hydrothermal pretreatment possessed a high specific surface area and a more complete K-PAC surface pore structure, which provided more adsorption sites for the adsorption of TC. However, the surface layer of F-PAC does not have the same complete pore structure as K-PAC. From the isotherm models, the adsorption process is more consistent with the statement of monolayer adsorption, and the influence of liquid film diffusion and intraparticle diffusion in the adsorption process can be imagined. From the kinetic analysis, it is clear that the PSO model fits the adsorption data more closely, so chemisorption occupies a pivotal position in the whole adsorption process. From the FTIR spectrogram, it can be seen that the PAC surface is rich in functional groups, including oxygen-containing [47] and hydroxyl groups, which can form robust hydrogen bonds with the amine group, phenol, and alkenone moieties of tetracycline [48]. Furthermore, activated carbon possesses C=C bonds, with one of the carriers of π-πEDA interactions being the carbon-carbon double bond [49]. Given that tetracycline is an electron-deficient system, carbon activated by KOH exhibits a robust electron-donating capacity [50], enabling TC to participate in the reaction as a π-electron acceptor. Considering the aforementioned analysis, the π-πEDA interaction also plays a significant role in the TC adsorption process by PAC [51]. From this, we can conclude that the adsorption process of TC on PAC prepared by hydrothermal pretreatment with KOH is mainly the result of pore filling and π-πEDA interactions.

## 4. Conclusions

In conclusion, high-performance PAC was successfully prepared by hydrothermal pretreatment. Experimental results showed that PAC with pinecone as precursor exhibited distinct characteristics, including a significantly high specific surface area (up to 1744.659 cm^2^/g) and a strong adsorption capacity of TC on its surface (up to 840.62 mg/g). Characterization analysis and isotherm modeling support the idea that pore filling is one of the main processes in TC adsorption. In addition, π-πEDA interactions were found to be another major adsorption mechanism through adsorption experiments and kinetic modeling analysis. The utilization of pinecones, an inexpensive and easily accessible material, in this research, presents an economically viable solution. The activated carbon produced through pretreatment demonstrates the promising potential for wastewater treatment, thereby highlighting PAC as a cost-effective and highly efficient adsorbent for TC removal from water.

## Figures and Tables

**Table 1:** Kinetic models and equations.

Kinetic Models	Equations	Simplified Equations
PFO	$\frac{dq_{t}}{dt}=k_{1}(q_{e}-q_{t})$	$\ln\left(q_{e}-q_{t}\right)=lnq_{e}-k_{1}t$
PSO	$\frac{dq_{t}}{dt}=k_{2}{\left(q_{e}-q_{t}\right)}^{2}$	$\frac{t}{q_{t}}=\frac{t}{q_{e}}+\frac{1}{k_{2}{q_{e}}^{2}}$
W-M	$q_{t}=k_{3}t^{\frac{1}{2}}+c$	
Boyd	$-\ln\left(1-R\right)=k_{4}t$	

**Table 2:** Isotherm models and equations.

Isotherm Models	Equations	Simplified Equations
Langmuir	$\frac{C_{e}}{q_{e}}=\frac{C_{e}}{q_{m}}+\frac{1}{k_{L}q_{m}}$	$q_{e}=k_{L}\frac{C_{e}q_{m}}{1+k_{L}C_{e}}$, $R_{L}=\frac{1}{1+K_{L}C_{0}}$
Freundlich	$lnq_{e}=lnk_{F}+\frac{1}{n}lnC_{e}$	$q_{e}=k_{F}{C_{e}}^{\frac{1}{n}}$
Temkin	$k_{T}C_{e}=e^{\frac{b_{T}q_{e}}{RT}}$	$q_{e}=RT\frac{1}{b_{T}}ln(k_{T}C_{e})$
R-P		$q_{e}=\frac{AC_{e}}{1+B{C_{e}}^{g}}$

Note: $R_{L}$ is the Langmuir isotherm dimensionless separation factor. When $R_{L}$ = 0, the adsorption is irreversible; when 0 < $R_{L}$ < 1 it is favorable adsorption; when $R_{L}$ > 1, it is unfavorable adsorption; the larger $k_{F}$, *n*, the better the adsorption performance of the adsorbent; when 0.1 ≤ $\frac{1}{n}$ ≤ 0.5, the adsorption is easy to occur; when $\frac{1}{n}$ > 2, the adsorption is harder to occur; when $b_{T}$ > 1, the adsorption process is an exothermic reaction; when $b_{T}$ < 1, the adsorption process is a heat absorption reaction; 0 < g < 1; the closer the value of g is to 1, the more suitable the Langmuir model is for describing the adsorption process.

**Table 3:** Specific surface area and pore structure of activated carbon.

Samples	Specific Surface Area (m^2^·g^−1^)	Total Hole Volume (cm^3^·g^−1^)	Average Pore Size (nm)	DFT Hole Volume (cm^3^·g^−1^)	HK Microporous Volume (cm^3^·g^−1^)
K6-PAC	1744.659	1.080	1.167	0.998	0.769
F6-PAC	1668.427	1.006	1.165	0.921	0.741

**Table 4:** Kinetic parameters of PAC.

	PAC	K6-PAC	F6-PAC
Kinetic Models			
PFO			
$k_{1}$		0.00275	0.00284
${qe}_{1}$		2152.962	2334.166
$R^{2}$		0.59022	0.56839
PSO			
$k_{2}$		0.0000053	0.0000056
${qe}_{2}$		833.333	815.4516
$R^{2}$		0.99312	0.98961
W-M			
$k_{3}$		4.64527	4.08544
c		472.91256	481.69209
$R^{2}$		0.98646	0.96885
Boyd			
$k_{4}$		1.82567 × 10^−4^	1.55997 × 10^−4^
$R^{2}$		0.99245	0.96118

**Table 5:** Isotherm parameters of PAC.

	PAC	K6-PAC	F6-PAC
Isotherm Models			
Langmuir			
$q_{m}$		833.3333	815.4516
$k_{L}$		0.92088	2.36153
$R^{2}$		0.97599	0.91892
Freundlich			
$k_{F}$		429.00876	460.55071
*n*		3.43607	5.07460
$R^{2}$		0.86336	0.79174
Temkin			
$b_{T}$		0.86826	0.79027
$k_{T}$		12.32002	67.58946
$R^{2}$		0.96682	0.91452
R-P			
A		758.68979	1730.56841
B		0.73178	2.13001
g		0.94905	0.91666
$R^{2}$		0.98506	0.94846

**Table 6:** Adsorption capacity of different adsorbents for TC.

Sorbents	Experimental Conditions	Maximum Adsorption Capacity (mg/g)	References
Zinc ferrite/chitosan-curdlan	Co-precipitation approach at 120 °C	371.40	[41]
Cobalt-impregnated biochar	NaOH impregnation and pyrolysis at 700 °C	370.37	[42]
Magnetic biochars	Pyrolysis accompanied by KOH activation	405.01	[43]
Chlorella biochar	Two-step pyrolysis at 800 °C	310.70	[44]
Mg-Al-LDOs-tailed biochar	One-pot co-precipitation at 600 °C	250.60	[45]
Pinecone-based activated carbon	KOH impregnation and two-step pyrolysis at 700 °C	840.62	This article

## Data Availability

Not applicable.
